# Antifungal metabolites of biocontrol stain LB-1 and their inhibition mechanism against *Botrytis cinerea*

**DOI:** 10.3389/fmicb.2024.1444996

**Published:** 2024-09-04

**Authors:** Huan Zhang, Hongliang Ji, Caiyun Liu

**Affiliations:** University Characteristic Laboratory of Precision Cultivation and Germplasm Innovation of Horticultural Crops in Shandong, School of Advanced Agricultural Sciences, Weifang University, Weifang, Shandong, China

**Keywords:** *Chaetomium subaffine*, antifungal substance, cellular structure, cell metabolism, proliferation

## Abstract

**Introduction:**

*Chaetomium subaffine* LB-1 is a novel biocontrol strain that produces non-volatile metabolites that inhibit the growth of *Botrytis cinerea*. However, the specific metabolites and antimicrobial mechanism of the strain LB-1 remains unclear.

**Methods:**

In this study, the antifungal substances produced by strain LB-1, as well as the underlying mechanism of its inhibitory effect against *B. cinerea*, were explored using metabolomic and transcriptomic analysis.

**Results:**

The results found that 45 metabolites might be the key antifungal substances, such as ouabain, ferulic acid, chlorogenic acid, spermidine, stachydrine, and stearic acid. The transcriptomic analysis indicated that the inhibition effect of LB-1 on *B. cinerea* resulted in the upregulation of genes related to adenosine triphosphate (ATP)-binding cassette (ABC) transporters, peroxisome, ER stress, and multiple metabolic pathways, and in downregulation of many genes associated with the synthesis of cell walls/membranes, carbohydrate metabolism, cell cycle, meiosis, and DNA replication.

**Discussion:**

These results suggested that the inhibitory effect of strain LB-1 against *B. cinerea* might be due to the destroyed cell wall and membrane integrity exerted by antimicrobial substances, which affect cell metabolism and inhibit cell proliferation.

## Introduction

In recent years, *Chaetomium* spp. have been demonstrated as potential biological control agents against plant diseases, and their main action mechanism on pathogenic fungi was the production of antimicrobial substances (Rao et al., 2023). Multiple reports have illustrated that *Chaetomium* spp. induced pathogen inhibition by secreting secondary metabolites (Rao et al., 2023). To date, many polyketides, such as chaetoviridin A, chaetoviridin B, chaetomugilin, and chaetoglobosin, have been identified from *Chaetomium globosum*, which can inhibit various phytopathogens, such as *Botrytis cinerea, Sclerotinia sclerotiorum, Fusarium graminearum, Phytophthora capsici, Verticillium dahliae*, and *Fusarium moniliforme* (Rao et al., 2023). In fact, in addition to these typical antimicrobial substances, various *Chaetomium* spp. contain numerous other specialized metabolites with biological activities including alkanones, pyrones, peptaibols, and gliotoxins, which also have different degrees of antimicrobial activities against pathogens (Rao et al., 2023; Darshan et al., 2021; Kumar et al., 2021). These specific metabolites in *Chaetomium* spp. have been continuously identified, and most researchers focused on analyzing the fungistatic active components in their fermentation broth using chemical extraction methods (Li et al., 2011; Zhao et al., 2021) and the research on antimicrobial substances involved in the fermentation of *Chaetomium* spp. using metabolomic profiling was little.

The inhibitory ability of antimicrobial substances might be related to the prevention of fungal conidial germination (Zhang et al., 2023a) and damage to membranes and/or cell walls (Wang et al., 2022a; Song et al., 2022), which caused metabolic disorders in cells (Wang et al., 2022b; Zhang et al., 2022). However, the inhibitory mechanism of biocontrol agents on pathogens might be the combination of several metabolites other than some specific metabolites. For example, the action mechanism of the chaetoviridin A in *C. globosum* has gradually been recognized, and it has been found to inhibit the cholesteryl ester transfer protein (CETP) in mice (Yan et al., 2018), to degrade the cell wall of *V. dahliae* (Zhang et al., 2021), and to inhibit the spore germination of *V. dahliae* by regulating genes related to cell membrane, amino acids, and sugar metabolism (Zhang et al., 2023b), but the underlying mechanism remains unclear. In fact, it is widely known that multiple antifungal metabolites were present in *C. globosum*, their mechanisms of inhibition were diverse, and their antifungal mechanisms were complex (Rao et al., 2023). Therefore, to explore the complex antifungal mechanisms with various metabolites, it is necessary to have a detailed understanding of the metabolic profile of *C. globosum* in the process of inhibiting pathogens and the molecular response mechanisms of pathogens.

*Chaetomium subaffine* LB-1 is a novel biocontrol strain screened by our research group, which was identified as an excellent antagonist against many common phytopathogenic fungi such as *B. cinerea, Exserohilum turcicum*, and *Bipolaris maydis* (Liu et al., 2021). Our previous study revealed that the inhibitory effect of LB-1 on phytopathogenic fungi involved multiple mechanisms (Liu et al., 2023a), in which producing extracellular non-volatile antifungal substances was important (Liu et al., 2023b). However, the specific compounds that contributed to the biocontrol effect of LB-1 and their inhibition mechanism remain unclear and need to be further researched. Thus, this study aimed to identify antifungal metabolites in LB-1 cell-free culture and understand the transcriptional alteration of genes in *B. cinerea* caused by LB-1.

## Materials and methods

### Strains and culture medium

Biocontrol strain LB-1 has been preserved in our laboratory since 2013 (Liu et al., 2015). *B. cinerea* B05.10 was purchased from the China Center for Type Culture Collection and stored in our laboratory, which was used as a test phytopathogenic fungus for experimental research. All aforementioned strains were cultured on potato dextrose agar medium (PDA, containing 20% filtered potato juice, 2% dextrose, and 1.8% agar) for 3 days (d) before being used in this research.

A 9-mm-diameter mycelial disc of strain LB-1 was inoculated in potato dextrose broth (PDB, containing 20% filtered potato juice, 2% dextrose) and shake culture (25°C, 130 r/min) for 10 days. The inoculation amount was one mycelial disc per 30-ml PDB. The shaking-cultured broth was filtered with three layers of sterile gauze, and the filtrate was centrifuged (8,000 *g*) for 15 min; the supernatant was used as LB-1 culture filtrate.

### The inhibitory effect observation of LB-1 culture filtrate on *B. cinerea*

The 9-mm-diameter mycelial discs of the test phytopathogenic fungus *B. cinerea* were inoculated in LB-1 culture filtrate and shaking-cultured (25°C, 130 r/min). Then, the morphological characteristics of hyphae were observed with a microscope at 24-, 48-, 72-, and 144-h postinoculation. Mycelial of *B. cinerea* cultured in PDB served as the corresponding control. Three replicates were performed for each sample.

### Metabolomics analyses

#### Sample preparation

Strain LB-1 and *B. cinerea* were cultured in PDB for 48 h, respectively, as two control groups (LB-1_Ck and B_Ck). The *B. cinerea* was shaking-cultured (25°C, 130 r/min) in LB-1 culture filtrate for 48 h as the experimental group (LB-1_B). Three groups of culture medium were sequentially filtered through gauze and microporous filter membranes, respectively. Finally, the filtrate from each group was divided into three new centrifuge tubes (5 ml) as biological replicates and freeze-dried to a dry powder for the detection of metabolites.

A 10-mg frozen powder was redissolved in a 200-μl methanol-water (8:2, v/v) solution and then centrifuged (15,000 *g*, 4°C) for 15 min. The supernatant was diluted to a final concentration containing 53% methanol by liquid chromatography–mass spectrometry (LC–MS) grade water and then centrifuged under the same parameters (15,000 *g*, 4°C, 15 min). The supernatant (50 μl) was collected and filtered with a polytetrafluoroethylene (PTFE) filter (0.22 μm) before being injected for ultraperformance liquid chromatography–mass spectrometry (UHPLC)–MS/mass spectrometry (MS) analysis.

#### LC–MS for untargeted metabolomics

Metabolite analysis was performed on the Vanquish UHPLC system coupled with the Orbitrap Q Exactive TM HF spectrometer system (ThermoFisher, Germany). The chromatographic column was Hypesil Gold C18 (100 mm × 2.1 mm, 1.9 μm; Thermo Fisher Scientific, Germany). The injection volume of the filtered supernatant was 10 μl, and the flow rate was 0.2 ml/min. The detection parameters were prepared as described previously (Lu et al., 2019). Here, the gradient for chromatographic analysis is displayed in Supplementary Table 1, and the setting parameters of LC–MS are shown in Supplementary Table 2.

#### Data processing

The chromatogram obtained from UHPLC–MS/MS was processed using Compound Discoverer 3.1 (CD3.1, Thermo Fisher Scientific), which was used for baseline filtering, peak pick-up, signal integration, retention time correction, and peak alignment based on certain parameters (Lu et al., 2019). Subsequently, the compound molecular weight, retention time, and peak area were exported in a data matrix, and peak intensities were normalized to the total spectral intensity. The normalized data were used to predict the molecular formula after searching the mzCloud (https://www.mzcloud.org/) and ChemSpider (http://www.chemspider.com/) databases to obtain the qualitative and quantitative results of metabolites in each sample. Kyoto Encyclopedia of Genes and Genomes (KEGG; https://www.genome.jp/kegg/pathway.html), HMDB (http://www.hmdb.ca/), and LIPID Maps (http://www.lipidmaps.org/) databases were used to annotate metabolites. Finally, metabolite data analysis was conducted with the principal component analysis (PCA; Wen et al., 2017), and univariate analysis (*t*-test) was used to calculate the statistical significance (*p*-value). The metabolites with Variable Importance in Projection (VIP) > 1, FoldChange (FC) > 1.5 or FC < 0.667, and a *p*-value of < 0.05 were considered differential metabolites (Heischmann et al., 2016). Pathway analysis of differential metabolites was conducted on the MetaboAnalystweb server (https://www.metaboanalyst.ca/).

### Transcriptomic analyses

#### Sample preparation

*B. cinerea* was shaking-cultured (25°C, 130 r/min) in LB-1 culture filtrate for 48 h (B_T), then the culture mycelia were washed with phosphate-buffered saline for 3 times, and used for RNA extraction. *B. cinerea* cultured in PDB for 48 h was used as a control (B_C).

#### Transcriptome sequencing

Total RNA was extracted using the Plant RNA Kit (Tiangen DP441, China) following the manufacturer's instructions. The concentration and integrity of RNA were tested using a NanoPhotometer spectrophotometer (IMPLEN, Germany) and RNA Nano 6000 Assay Kit of the Bioanalyzer 5400 system (Agilent, USA), respectively. Extracted RNA was used for library construction with the NEBNext Ultra RNA library Prep kit from Illumina (NEB, USA) and sequenced on an Illumina NovaSeq 6000 platform (Illumina, USA) with a paired-end 150 mode. Library preparation and high-throughput sequencing were performed using instruments from Novogene (Beijing, China).

#### Transcriptome data analysis

All raw reads of RNA sequencing (RNA-seq) data (NCBI: PRJNA953188) were filtered and trimmed using FASTP (version 0.19.7; Chen et al., 2018), and the high-quality reads were obtained and mapped to the genome of *B. cinerea* B05.10 (Staats and van Kan, 2012) using HISAT2 (version 2.0.5) software (Mortazavi et al., 2008). The expression was then standardized by fragments per kilobases per million reads (FPKM; Bray et al., 2016). The differential expression analysis was carried out using the DESeq R package; genes with |log_2_(FoldChange)| ≥1.5 and adjusted *p*-values of ≤ 0.05 were regarded as differentially expressed (Love et al., 2014). Afterward, differentially expressed genes (DEGs) were subjected to KEGG pathway enrichment analysis using clusterProfiler (version 3.4.4) software (Yu et al., 2012). The annotation of gene functions was carried out using the reference genome, swiss prot sequence protein, and the homologous protein family (Pfam) database.

#### Quantitative real-time polymerase chain reaction (qRT-PCR)

To validate the reliability of transcriptome sequencing data, taking the *actin* gene as an internal reference (Staats and van Kan, 2012), 15 genes were selected to perform qRT-PCR analysis using 7500 Fast Real-Time PCR System (ThermoFisher, Germany). Genes were selected based on expression fold change in the two culture filtrates and also based on their biological functions. The relative gene expression levels were analyzed using the 2^−ΔΔT^ method (Livak and Schmittgen, 2001). Each sample group was subjected to three biological replications. The primers were designed using Primer5 and are presented in Supplementary Table 3.

### Statistical analysis

All experiments were repeated in triplicate. Quantitative data on 20 genes were subjected to analysis of variance (ANOVA) using Statistical Package for the Social Sciences (SPSS) software (version 19.0, IBM, NY, USA). Data were shown as mean values ± standard errors, and *p* < 0.05 was considered statistically significant by the Student's *t*-test.

## Results

### Effects of LB-1 cell-free culture broth on the morphological characteristics of *B. cinerea*

**Figure 1:**
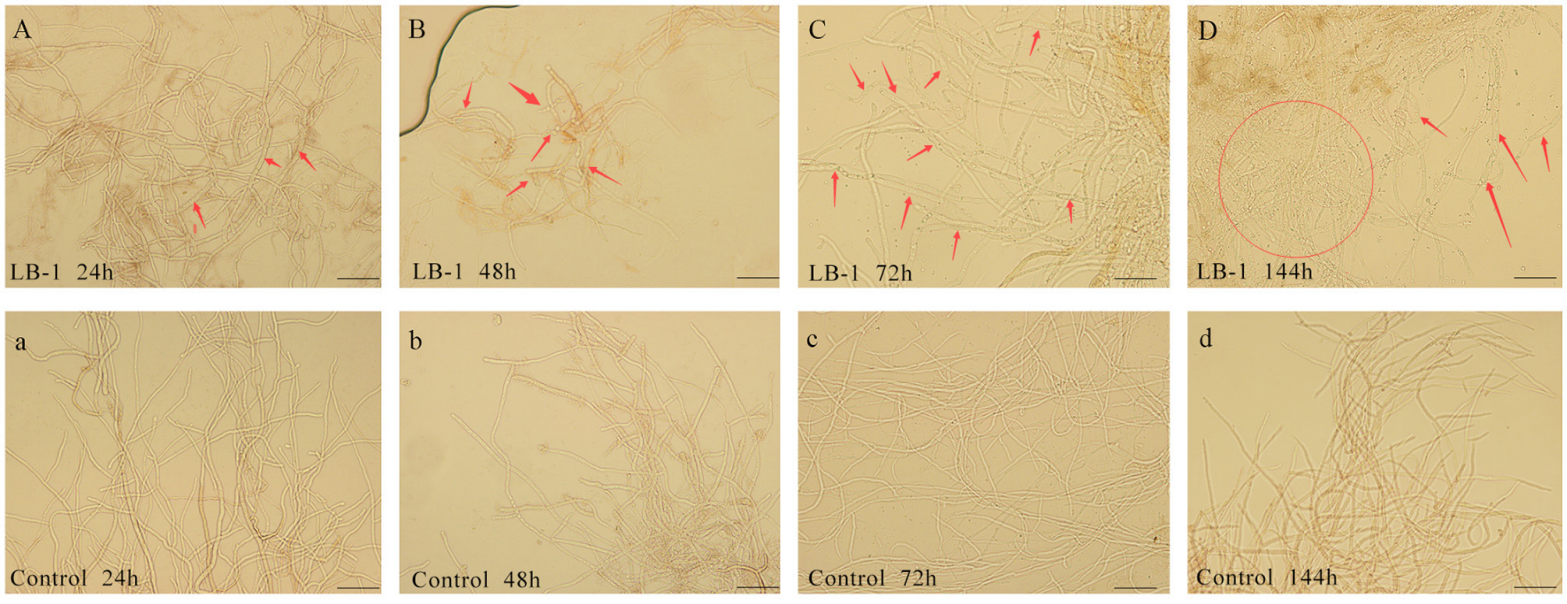
The effect of LB-1 on the morphology of *Botrytis cinerea* mycelia. Mycelia of *B. cinerea* cultured in LB-1 cell-free culture broth **(A)**, exhibited swelling at 48 h **(B)**, and then vacuolated and aggravated **(C, D)**, while *B. cinerea* cultured in control potato dextrose broth (PDB) kept normal smooth tubular morphology constantly at 24 h **(a)**, 48 h **(b)**, 72 h **(c)**, and 144 h **(d)**. Scale bar = 50 μm.

Microscopic observation revealed that compared to a control group treated with PDB culture medium (Figure 1A), the *B. cinerea* cultured in LB-1 cell-free culture broth maintained a normal morphology similar to the control (Figure 1A). However, after 48 h, the treated mycelia of *B. cinerea* exhibited wrinkling and deformities (Figure 1B). Furthermore, continuous exposure to LB-1 culture broth for 72 h and 144 h resulted in aggravated abnormal swelling morphological characteristics of the treated mycelia (Figures 1C, D), while the control mycelia of *B. cinerea* remained normal (Figures 1C, D). These findings suggested that certain compounds or enzymes present in the LB-1 cell-free culture broth may have an impact on the normal growth and development of *B. cinerea*.

### The identification of LB-1 antifungal metabolites through metabolomics analysis

**Figure 2:**
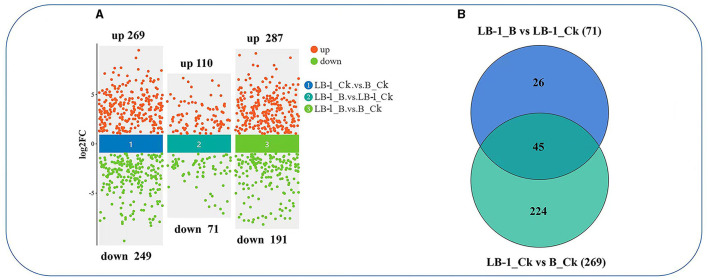
The differential metabolites in the process of LB-1 against *Botrytis cinerea*. **(A)** Volcano diagrams of the differential metabolites in the three compared combinations. The *y*-axis shows log_2_(FoldChange) and the *x*-axis shows comparison group (① LB-1_Ck vs. B_Ck, ② LB-1_B vs. LB-1_Ck, and ③ LB-1_B vs. B_Ck), Each point in the figure represents a metabolite. **(B)** Venn diagram analysis of unique/shared metabolites among the two compared combinations. The blue circle represents the metabolite sets in LB-1_B vs. LB-1_Ck, and the green circle represents the metabolite sets in LB-1_Ck vs. B_Ck.

To investigate the antifungal metabolites produced by strain LB-1 involved in the biocontrol of *B. cinerea*, we conducted an untargeted metabolomic analysis of LB-1 cell-free culture broth in the presence (inoculated with *B. cinerea* for 48 h; sample name: LB-1_B) and absence of *B. cinerea* (LB-1_Ck). We also analyzed the extracellular metabolites of *B. cinerea* (B_Ck). Initially, statistical analysis was performed to identify differential metabolites (FC > 1.5 or FC < 0.667; *p*-value < 0.05) in each pairwise comparison (LB-1_Ck vs. B_Ck, LB-1_B vs. LB-1_Ck, and LB-1_B vs. B_Ck; Figure 2A). The details of metabolites, FC, and *p*-values for each significantly altered metabolite in the three comparative combinations were provided in Supplementary Tables 1–3, respectively.

The *B. cinerea* cultured in LB-1 cell-free culture broth exhibited significant metabolic changes (LB-1_B vs. B_Ck), with 287 metabolites significantly upregulated and 191 significantly downregulated (Supplementary Table 3; Figure 2A). A total of 515 metabolites showed significant differences between LB-1_Ck and B_Ck (Supplementary Table 1; Figure 2A). Notably, 269 metabolites were significantly higher in LB-1 than in *B. cinerea* (LB-1_Ck vs. B_Ck), suggesting the presence of potential antifungal metabolites among them. After inoculation with *B. cinerea* for 48 h, 181 metabolites in the LB-1 cell-free culture broth exhibited differences, 110 metabolites were upregulated, and 71 metabolites were downregulated (LB-1_B vs. LB-1_Ck) (Supplementary Table 2; Figure 2A). Most antifungal metabolites secreted by the biocontrol strain are consumed after acting on pathogenic fungi. Therefore, these 71 metabolites were considered to be the possible antifungal metabolites of strain LB-1, which could be mainly classified into nine categories, namely, 16 nucleotides, 14 glycerophospholipids, nine alkaloids, nine carbohydrates, six peptide compounds, five phenolic compounds, four fatty acids, two ketone compounds, and six others (Supplementary Figure 1).

Interestingly, 45 metabolites in the LB-1 cell-free culture broth decreased after inoculation with *B. cinerea* (LB-1_B vs. LB-1_Ck), and these 45 metabolites secreted were originally significantly higher than those of *B. cinerea* (LB-1_Ck vs. B_Ck; Figure 2B; Table 1; Supplementary Table 4). Thus, these 45 metabolites were considered to be the key antifungal metabolites of strain LB-1. These 45 metabolites included 13 nucleotides such as guanosine monophosphate, deoxyadenosine 5′-monophosphate (dAMP), guanosine monophosphate (GMP), 3′-adenosine monophosphate (3′-AMP), and adenosine 5′-monophosphate, and 7 glycerophospholipids, such as lysophosphatidylethanolamine (LPE) 18:0, LPE 16:0, phosphatidylcholine (PC) (14:0e/2:0), and lysophosphatidylcholine (LPC) 18:1. There were also six alkaloids, namely, 1-acetyl-*N*-(6-chloro-1,3-benzothiazol-2-yl)-4-piperidinecarboxamide, DL-stachydrine, spermidine, PB-22 *N*-pentanoic acid-3-carboxyindole, tert-butyl *N*-[1-(aminocarbonyl)-3-methylbutyl]carbamate, and 1-allyl-4,5-diphenyl-2-(2-thienyl)-1H-imidazole, and five phenolic compounds, namely, ferulic acid, chlorogenic acid, ouabain, 1-caffeoylquinic acid, and 23-nordeoxycholic acid. Additionally, there were four fatty acids (16-hydroxyhexadecanoic acid, fatty acid esters of hydroxy fatty acids (FAHFA) [16:0/18:2], stearic acid, FAHFA [18:0/20:2]), four carbohydrates (D-ribulose 1,5-bisphosphate, 6-phosphogluconic acid, D-sedoheptulose 7-phosphate, benzyl 6-*O*-β-D-glucopyranosyl-β-D-glucopyranoside), and two peptide compounds (glutathione [GSH; reduced], L-glutathione oxidized), as well as one ketone compound (methyltestosterone) and three other compounds (mag [18:1], ethyl 4-hydroxy-2-phenylquinoline-6-carboxylate, metabolite (WLH); Table 1). Further evaluation is needed to determine the antifungal effects and biocontrol potential of these specific metabolites against pathogens.

**Table 1:** Forty-five key antifungal metabolites in the cell-free culture broth of LB-1.

**Category**	**Compound name**	**Formula**	**Molecular weight**	**Mass-to-charge ratio (m/z)**	**log_2_(FoldChange) LB-1_Ck vs. B_Ck**	**log_2_(FoldChange) LB-1_B vs. LB-1_Ck**
Alkaloids	1-Acetyl-*N*-(6-chloro-1,3-benzothiazol-2-yl)-4-piperidinecarboxamide	C_15_ H_16_ Cl N_3_ O_2_ S	337.06752	364.06662	4.93	−5.85
	DL-Stachydrine	C_7_ H_13_ N O_2_	143.09538	522.35712	2.23	−1.52
	Spermidine	C_7_ H_19_ N_3_	128.13208	291.06546	4.59	−2.33
	PB-22 *N*-pentanoic acid-3-carboxyindole	C_14_ H_15_ N O_4_	261.09576	324.06039	3.92	−2.51
	tert-butyl *N*-[1-(aminocarbonyl)-3-methylbutyl]carbamate	C_11_ H_22_ N_2_ O_3_	230.16417	308.97855	3.36	−1.55
	1-allyl-4,5-diphenyl-2-(2-thienyl)-1H-imidazole	C_22_ H_18_ N_2_ S	342.12307	332.07587	2.52	−1.80
Carbohydrates	D-Ribulose 1,5-bisphosphate	C_5_ H_12_ O_11_ P_2_	309.98583	193.05016	2.08	−2.28
	6-Phosphogluconic acid	C_6_ H_13_ O_10_ P	276.0248	336.06021	3.78	−2.79
	D-Sedoheptulose 7-phosphate	C_7_ H_15_ O_10_ P	290.04053	362.05057	4.83	−5.32
	Benzyl 6-*O*-β-D-glucopyranosyl-β-D-glucopyranoside	C_19_ H_28_ O_11_	432.16307	275.01752	4.40	−3.00
Fatty acids	16-Hydroxyhexadecanoic acid	C_16_ H_32_ O_3_	272.23505	348.0715	2.46	−2.18
	Fatty acid esters of hydroxy fatty acids (FAHFA) (16:0/18:2)	C_34_ H_62_ O_4_	534.46269	144.10266	2.87	−2.10
	Stearic acid	C_18_ H_36_ O_2_	284.27143	508.34094	2.72	−1.56
	FAHFA (18:0/20:2)	C_38_ H_70_ O_4_	590.52547	325.04413	4.20	−3.26
Glycerophospholipids	Lysopc 17:0	C_25_ H_52_ N O_7_ P	509.34823	353.08786	3.83	−2.91
	Lysophosphatidylcholine (LPC) 18:0	C_26_ H_54_ N O_7_ P	569.36951	294.11319	4.09	−3.03
	Lysophosphatidylethanolamine (LPE) 16:0	C_21_ H_44_ N O_7_ P	453.28653	568.3623	3.20	−1.78
	LPE 18:0	C_23_ H_48_ N O_7_ P	481.31698	346.05569	2.62	−1.91
	Phosphatidylcholine (PC) (14:0e/2:0)	C_24_ H_50_ N O_7_ P	495.33377	344.0401	2.76	−1.70
	PC (16:1e/2:0)	C_26_ H_52_ N _O7_ P	521.3497	129.13942	3.68	−1.84
	LPC 18:1	C_26_ H_52_ N O_7_ P	567.35438	322.04434	2.92	−1.24
Nucleotides	Guanosine monophosphate	C_10_ H_14_ N_5_ O_8_ P	363.05915	260.08853	6.35	−6.27
	Cytidine 5′-monophosphate (hydrate)	C_9_ H_14_ N_3_ O_8_ P	323.05319	152.05736	3.09	−3.36
	deoxyadenosine 5′-monophosphate (dAMP)	C_10_ H_14_ N_5_ O_6_ P	331.06877	346.05569	4.03	−3.79
	Guanosine monophosphate (GMP)	C_10_ H_14_ N_5_ O_8_ P	363.05789	231.17159	6.35	−7.06
	3′-Adenosine monophosphate (3′-AMP)	C_10_ H_14_ N_5_ O_7_ P	347.06416	289.03317	6.88	−5.75
	UMP	C_9_ H_13_ N_2_ O_9_ P	324.037	629.28571	3.65	−2.17
	Adenosine 5′-monophosphate	C_10_ H_14_ N_5_ O_7_ P	347.06295	454.29373	6.02	−6.39
	Guanosine-3′,5′-cyclic monophosphate	C_10_ H_12_ N_5_ O_7_ P	345.04745	480.30978	5.21	−4.25
	Cytidine-5′-monophosphate	C_9_ H_14_ N_3_ O_8_ P	323.05163	477.21896	4.35	−4.42
	Guanine	C5 H5 N5 O	151.05005	301.21704	7.42	−3.24
	Guanosine 3′,5′-cyclic monophosphate	C H_4_ N_2_ O	345.04843	533.45514	5.21	−3.10
	Deoxycytidine 5′-monophosphate	C_9_ H_14_ N_3_ O_7_ P	307.08461	496.34067	3.54	−1.81
	Xanthosine	C_10_ H_12_ N_4_ O_6_	284.07567	613.1615	2.87	−1.72
Peptide compounds	Glutathione (reduced)	C_10_ H_17_ N_3_ O_6_ S	290.05814	431.15567	2.44	−2.24
	L-Glutathione oxidized	C_20_ H_32_ N_6_ O_12_ S_2_	612.15394	355.10361	2.44	−2.36
Phenolic compounds	Chlorogenic acid	C_16_ H_18_ O_9_	354.09526	566.34711	2.23	−2.15
	Ferulic acid	C_10_ H_10_ O_4_	194.05745	308.09177	3.11	−0.80
	Ouabain	C_29_ H_44_ O_12_	630.29283	283.26413	4.43	−2.00
	1-Caffeoylquinic acid	C_16_ H_18_ O_9_	354.09635	589.51849	1.28	−1.10
	23-Nordeoxycholic acid	C_23_ H_38_ O_4_	378.27536	379.28244	2.32	−1.73
Ketone compounds	Methyltestosterone	C_20_ H_30_ O_2_	302.22433	271.22781	2.93	−2.02
Other	Mag (18:1)	C_21_ H_40_ O_4_	356.29347	357.30096	2.17	−1.56
	ethyl 4-hydroxy-2-phenylquinoline-6-carboxylate	C_18_ H_15_ N O_3_	293.10603	283.06839	3.64	−3.10
	WLH	C_23_ H_30_ N_6_ O_4_	476.21147	343.1304	6.37	−2.87

### Transcriptome alteration in *B. cinerea* induced by antifungal compounds

#### Sequencing and mapping

Using RNA-Seq, 4.15–5.11 million raw reads were generated from the six libraries (Table 2). After filtering out adaptor sequences and removing low-quality reads, we obtained 4.02–4.99 million clean reads, with a Q20 percentage (an error probability of 0.03) of over 96% (Table 2). These clean reads were then mapped to the *B. cinerea* B05.10 genome, with ~3.89–4.83 million clean reads successfully mapped, accounting for 96.88–96.72% of the total reads (Table 2). These results indicated that the sequencing quality and quantity were sufficient for further analysis. Additionally, the correlation diagram of gene expression levels between samples displayed high correlations (Pearson's *r* > 0.90) within biological replicates (Supplementary Figure 2), suggesting the good repeatability of this experiment.

**Table 2:** A summary statistics of clean reads in the *B. cinerea* transcriptomes.

**Sample**	**Raw reads (bases)**	**Clean reads (bases)**	**Uniquely mapped**	**Guanine-cytosine(GC) content (%)**	**Error rate (%)**	**Q20 (%)**	**Total mapped**
B_T-1	51,185,282 (7.68G)	49,939,148 (7.49G)	48,054,087 (96.23%)	47.25	0.03	96.91	48,300,366 (96.72%)
B_T-2	47,024,264 (7.05G)	45,663,610 (6.85G)	43,883,167 (96.1%)	47.11	0.03	96.84	44,121,746 (96.62%)
B_T-3	45,830,236 (6.87G)	44,518,456 (6.68G)	42,957,204 (96.49%)	47.07	0.03	96.97	43,167,832 (96.97%)
B_C-1	42,027,208 (6.3G)	40,870,756 (6.13G)	39,273,501 (96.09%)	47.02	0.03	96.24	39,492,653 (96.63%)
B_C-2	41,552,250 (6.23G)	40,206,706 (6.03G)	38,773,125 (96.43%)	47.09	0.03	96.74	38,952,664 (96.88%)
B_C-3	42,416,826 (6.36G)	40,242,444 (6.04G)	38,723,244 (96.22%)	46.86	0.03	96.86	38,943,531 (96.77%)

#### Transcriptional analysis of differential gene expression

**Figure 3:**
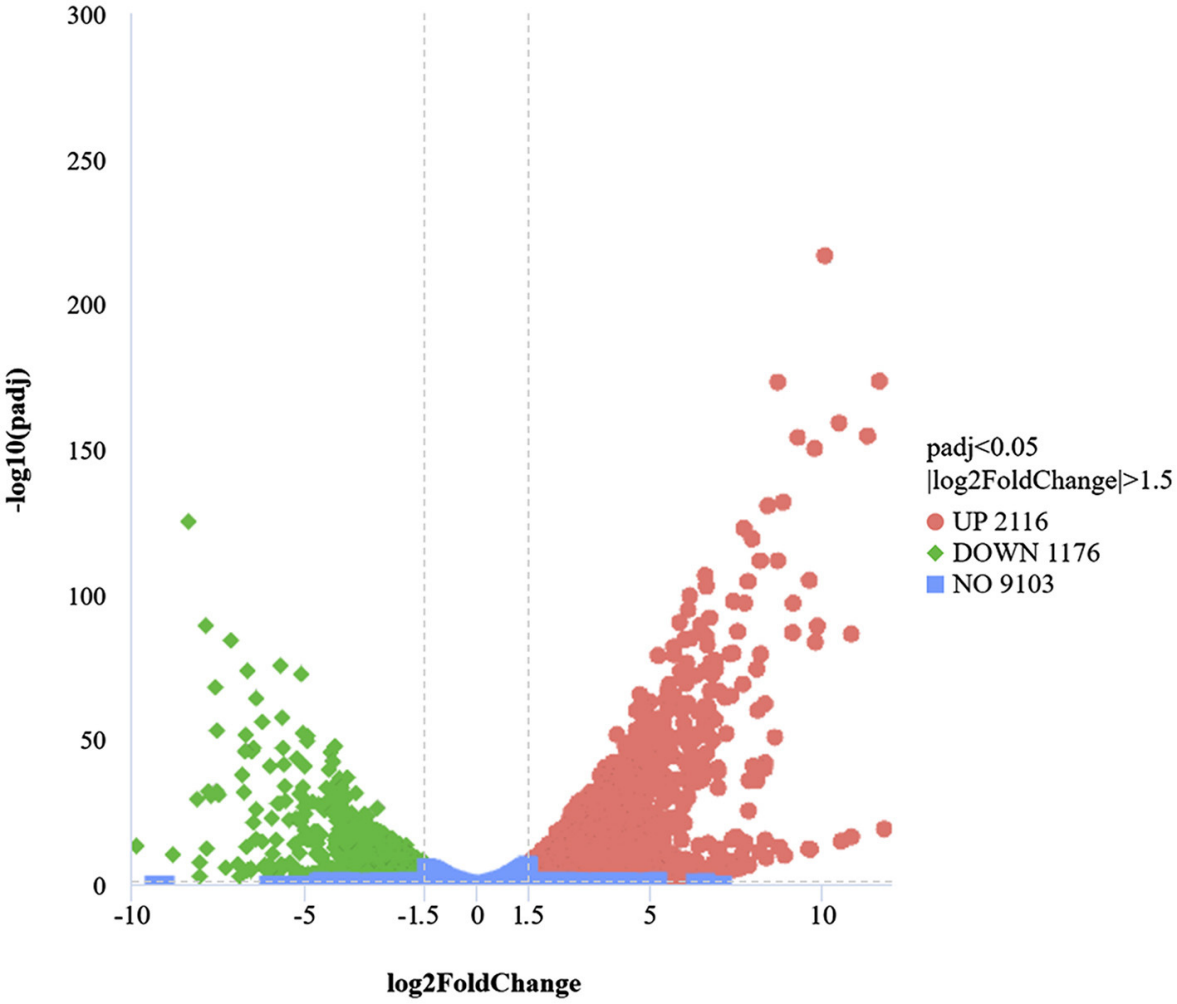
Analysis of differentially expressed genes (DEGs) in B_T and B_C. In volcano diagrams, the *x*-axis shows the fold change (log_2_[FoldChange]) in the expression of the genes in B_T vs. B_C, and the *y*-axis shows the significance level (–log_10_padj). The vertical lines show thresholds for a log2 ratio larger than 1.5 or lower than −1.5. The horizontal line shows the threshold for *p* < 0.05. The upregulated and downregulated genes are indicated by red and green dots, respectively.

A transcriptome analysis was conducted to identify the DEGs associated with the defense mechanisms of *B. cinerea* under LB-1 stress. A total of 3,292 DEGs were identified |log_2_(FoldChange)|≥1.5 and adj.*p*.Val ≤ 0.05) between B_T and B_C samples, consisting of 2,116 upregulated and 1,176 downregulated genes (Figure 3; Supplementary Table 5). Among these DEGs, some genes with significant fold changes in the volcanic map have caught our attention. We focused on the top 20 upregulated genes based on the log_2_FC values (log_2_FC > 6.6). Apart from 10 unannotated genes (*BCIN_14g01430, BCIN_01g03270, Bccpd1, BCIN_15g04700, BCIN_01g04930, BCIN_15g00320, BCIN_01g04580, BCIN_08g04600, BCIN_11g01080*, and *novel.399*), many defense-related genes were found, such as major facilitator superfamily multidrug transporter NAG4 (*gene-BCIN_02g07440*, log_2_FC = 12.37*)*, zinc-type alcohol dehydrogenase-like protein (*BCIN_05g08390*, log_2_FC = 11.63*; BCIN_10g01470*, log_2_FC = 11.29), plasma-membrane choline transporter (*Bcpie2*, log_2_FC = 8.69), sterol regulatory element-binding protein 1 (*BCIN_08g03080*, log_2_FC = 6.62), and heat shock protein (*BCIN_01g09530*, log_2_FC = 9.60). However, among the top downregulated 20 genes (log_2_FC < −6.7), except for 10 unannotated genes (*BCIN_05g01970, BCIN_05g02000, BCIN_05g02020, BCIN_05g01990, BCIN_05g02010, BCIN_08g00940, BcekdA, BCIN_06g01620, BCIN_05g02040*, and *BCIN_12g06760*), 6 hydrolases enzymes related to cell wall degradation have been discovered, including general α-glucoside permease (*BCIN_05g02030*, log_2_FC = −7.57), raucaffricine-o-β-d-glucosidase (*BCIN_16g03980*, log_2_FC = −4.93), glucan 1,3-β-glucosidase (*BCIN_05g01660*, log_2_FC = −4.13), glycosyl transferase family group 2 (*BCIN_06g03790*, log_2_FC = −5.65), cys-gly metallodipeptidase dug1 (*Bcdug2*, log_2_FC = −5.71), and neutral protease 2 (*BCIN_12g06300*, log_2_FC = −6.22). These results suggest that *B. cinerea* may alleviate LB-1 stress by activating a series of specific defense responses and adjusting cell wall degradation processes.

#### The identification of altered pathways-based KEGG enrichment analysis

**Figure 4:**
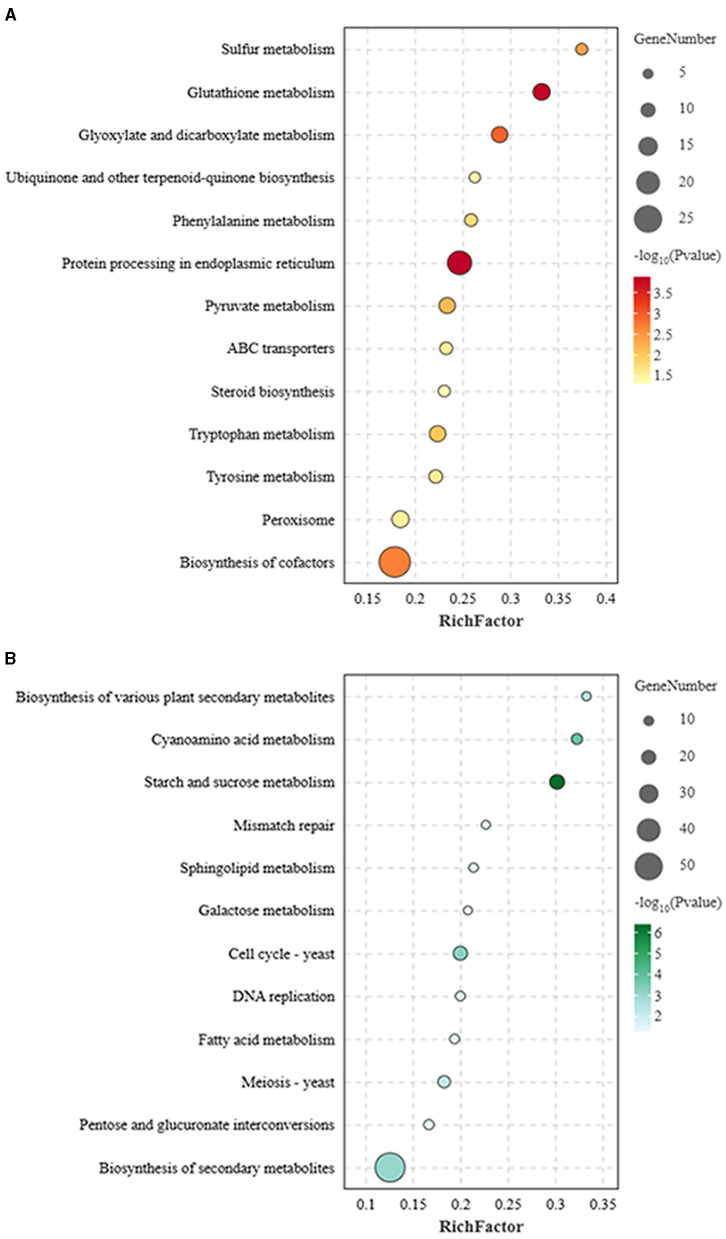
Kyoto Encyclopedia of Genes and Genomes (KEGG) pathway enrichment analysis of differentially expressed genes (DEGs). **(A)** KEGG pathways enriched for the upregulated *genes* in *Botrytis cinerea*. **(B)** KEGG pathways enriched for the downregulated *genes* in *B. cinerea*.

The KEGG analysis confirmed that the LB-1 modified the DEGs involved in multiple metabolism pathways in *B. cinerea*. Some defense-related pathways with predicted antifungal activities were significantly upregulated in this study (*p* < 0.05), including protein processing in the endoplasmic reticulum (ER), peroxisome, GSH metabolism, adenosine triphosphate (ATP)-binding cassette (ABC) transporters, and amino acids metabolism (Figure 4A; Supplementary Table 6). The significant enrichment pathway of downregulated genes did not involve these defense pathways but mainly enriched in some molecular function and biological processes, including cell cycle, meiosis, and DNA replication followed by carbohydrate metabolism (Figure 4B; Supplementary Table 7).

From these overexpressed pathways, it could be seen that multiple ABC transporters (ABC superfamily transporters) related to multidrug resistance were upregulated, including three subfamilies: ABCB (gene_name: *BCIN_01g03740, BCIN_16g03560*, and *BCIN_12g00700*), ABCC (*BCIN_01g00180*), and ABCG (*Bmr5, BcatrA*, and *Bcpdr11*; Supplementary Table 4). Meanwhile, the transport of matrix protein from cytoplasm to peroxisome was stimulated, in which the key docking complex protein PEX13 (*Bcpex13*) was upregulated (Supplementary Table 5), and a series of biochemical metabolic processes were activated, including overexpression of the stress-related enzymes catalase (CAT) (*Bccat5, BccatA*, and *Bccat6*) in the antioxidant system (Supplementary Table 5). In addition to the peroxisome, the enzymes related to scavenging reactive oxygen species (ROS) in GSH metabolism were upregulated, such as glutathione-S-transferase (GST) (*Bcgst3, Bcgst14, Bcgst21, Bcgst5, Bcgst1, Bcgst22, Bcgst26*, and *Bcgst6*; Supplementary Table 6), and the resistance gene, tyrosinase (*BCIN_06g00420*) in tyrosine metabolism, was also activated.

Strain LB-1 also caused endoplasmic reticulum stress (ERS) in *B. cinerea*. In the protein processing in the ER pathways, 2 genes (*Bcsec62* and *Bcero1*) related to correct or misfolded protein processing and 14 genes (*BCIN_02g04920, BCIN_15g04190, BCIN_15g02550, Bcubc6, Bcfes1, Bcotu1, BCIN_10g00300, Bcufd2, BCIN_03g09290, Bcsec63, Bcdsk2, Bcrad23, Bcpng1*, and *BCIN_08g04560*) from the ER-associated degradation (ERAD) were significantly upregulated in our study (Supplementary Table 7). Furthermore, the IRE1 signaling pathway on the ER membrane triggered by ERS receptor protein-IRE1 (*Bcire1*) was activated. Meanwhile, strain LB-1 triggers overexpression of some basic physiological metabolic pathways in *B. cinerea*, such as amino acid metabolism (bfu00380 and bfu00350), phenylalanine (bfu00360), and pyruvate (bfu00620) metabolism, as well as biosynthesis of cofactors (bfu01240; Figure 4A, Supplementary Table 6).

In addition to significantly activating these defense-related pathways, multiple growth and development-related pathways in *B. cinerea* were significantly inhibited (Supplementary Table 7; Figure 4B). For example, carbohydrate metabolism pathways, pentose (bfu00040) and galactose metabolism (bfu00052), and some enzyme genes regulating D-glucose and D-galactose in these pathways were found to be downregulated, including α-galactosidase (*BCIN_03g02710*) and α-glucosidase (*BCIN_11g06440*; Figure 4B), indicating that LB-1 had an inhibitory effect on energy metabolism in the mycelia of *B. cinerea*. Meanwhile, the pathways related to cell wall synthesis, such as various types of fatty acid metabolism (bfu01212), starch and sucrose metabolism (bfu00500), and the metabolic pathways of sphingolipids (bfu00600) related to maintaining the cell membrane structure were significantly downregulated, indicating that the function of the cell wall and membranes was hindered. It is worth noting that the DNA replication process (bfu03030) in *B. cinerea* is hindered, and the ability of mismatch repairs (bfu03430) was reduced, making damaged DNA unable to be repaired properly. In addition, many cyclins (*Bccks1*, and *Bccdc28*) and cell division (*Bcbub2* and *BCIN_14g00280*) control proteins were significantly downregulated, resulting in the inability to initiate cell division processes (Supplementary Table 8). The inhibition of cell development and proliferation was the main factor promoting apoptosis, suggesting that the growth of *B. cinerea* was affected.

### qRT-PCR validation

**Figure 5:**
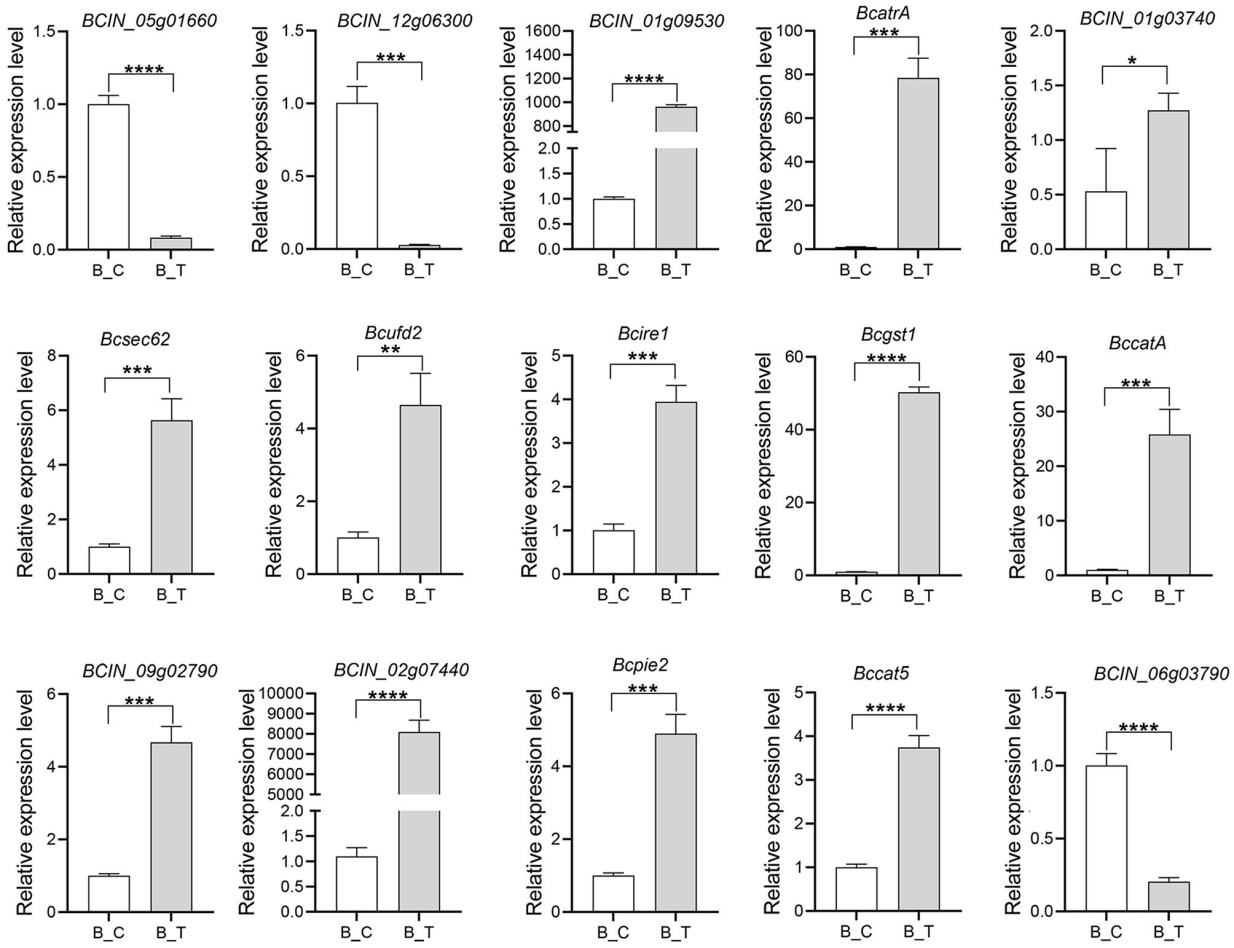
Quantitative real-time polymerase chain reaction (qRT-PCR) validation result of the 15 differentially expressed genes (DEGs). Error bars indicate the standard error of the mean (*n* = 3). Asterisks indicate a statistically significant difference based on the Student's *t*-test (*p < 0.05; **p < 0.01; ***p < 0.001; ****p < 0.0001).

To validate the DEG revealed by RNA-seq data, the level and patterns of the expression of the 15 genes involved in the inhibitory effects of strain LB-1 on *B. cinerea* were assessed by qRT-PCR (Figure 5). The genes included those encoding enzymes associated with the cell wall synthesis (*BCIN_12g06300, BCIN_05g01660*, and *BCIN_06g03790*), ABC transporters (*BCIN_01g03740*, and *BcatrA*), ER stress (*Bcsec62, Bcufd2*, and *Bcire1*), and peroxisomal (*Bccat5*, and *BccatA*), as well as some resistance genes (*Bcgst1, BCIN_09g02790, BCIN_02g07440, Bcpie2*, and *BCIN_01g09530*). The results showed that the pattern of expression of all the genes was similar between the RNA-seq and qRT-PCR results (Figure 5), suggesting that our transcriptome results were reliable.

## Discussion

Many research studies have proved that *Cheatomium* spp. were promising in plant disease control, and the bioactive constituent contained in their culture fermentation was a valuable research field for its utilization (Rao et al., 2023). Currently, the antimicrobial substance from *C. globosum* has been researched and identified extensively (Rao et al., 2023; Darshan et al., 2021; Kumar et al., 2021). *C. subaffine* LB-1 was a novel biocontrol strain discovered by our research group, which could obtain its inhibitory effect on phytopahtogenic fungi of *B. cinerea, B. maydis*, and *Alternaria solani*, etc., by non-volatile metabolites (Liu et al., 2023b) secreted in the culture broth. However, the specific metabolites and the related inhibition mechanism remained unclear. In this study, we confirmed that the cell-free culture broth of LB-1 exhibited considerable antifungal effect against *B. cinerea* (Figure 1). Thus, the exact antimicrobial substance contained in the cell-free culture broth of LB-1 was further researched by metabolic analysis of the genes, and the related metabolic pathway with its antifungal effect on *B. cinerea* was researched with transcriptomic analysis.

Through comprehensive analysis of the metabolites in LB-1 cell-free culture broth by untargeted metabolomics, the results showed that 71 metabolites were considered to be potential antifungal metabolites (Supplementary Figure 1, Supplementary Table 4). These metabolites are classified into different chemical categories (Supplementary Figure 1); of which, alkaloids, phenolic compounds, fatty acids, ketone compounds, and peptide compounds have been reported to have antioxidant, anticancer, antibiotic, and antiviral properties (Wang et al., 2022b; Rao et al., 2023). Moreover, 45 key antifungal substances (Table 1) were found among 71 metabolites, including caffeoylquinic acid (Liu et al., 2020), ouabain (Dell'Anno et al., 2022), ferulic acid (Yan et al., 2023), chlorogenic acid (da Silva et al., 2022), spermidine (Alves da Costa Ribeiro Quintans et al., 2022), stachydrine (Arrieche et al., 2022), and stearic acid (Bhaskaran et al., 2022), which have been described in relation to antimicrobial effects. Among these antimicrobial substances, 0.085% (m/v) ferulic acid (Patzke and Schieber, 2018) and 3 g/L chlorogenic acid (Zhang et al., 2023c) were identified as highly effective against the growth of *B. cinerea*. To our knowledge, the antifungal effect of other metabolites against *B. cinerea* has not been assessed. For example, spermidine, as a potent antifungal agent, with an EC_50_ of 21 μg/ml, can effectively inhibit *Alternaria* spp (Alves da Costa Ribeiro Quintans et al., 2022) and its inhibitory effect on *B. cinerea* is worth further investigation. Here, we suggested that these metabolites might contribute mainly to the antifungal effect of LB-1. However, more research, such as targeted chemical separation and purification as well as antifungal activity, is needed to elucidate it.

Antifungal substances could disrupt the structure of the cell membrane and cell wall in pathogens, interfere with their normal physiological metabolism, and thus inhibit their growth (Zhang et al., 2023a; Wang et al., 2022a; Song et al., 2022). In this study, the normal morphology and growth of *B. cinerea* were significantly disrupted and inhibited (Supplementary Figure 1). Based on transcriptome and qRT-PCR analysis, some genes associated with cell wall biosynthesis, such as neutral protease 2 (*BCIN_12g06300*), glucan 1,3-β-glucosidase (*BCIN_05g01660*), glycosyl transferase family group 2 (*BCIN_06g03790*), were downregulated by strain LB-1 (Supplementary Table 5; Figure 4). Meanwhile, the expression of cell-membrane-related genes was inhibited by several pathways involved in sphingolipid metabolism (Figure 3C). Sphingolipids and ergosterol are two important components of the cell membrane, which play a crucial role in maintaining membrane fluidity and stabilizing membrane structure (Mukhopadhyay et al., 2004). Wang et al. found that lauric acid mainly disrupts the structure of cell membranes by blocking the synthesis pathway of ergosterol, thereby accelerating the cell death of *Rhizoctonia solani* (Wang et al., 2022b). The cell wall/membrane was a target for antifungal substances to inactivate pathogens (Hasim and Coleman, 2019). Phenolic compounds were reported to disrupt the formation and assembly of cell walls by targeting the pathogen's cell wall (Zhang et al., 2023b) or disrupting the integrity of the cell membrane, leading to infiltration and inhibition of cell growth due to their strong hydrophobicity (Ma et al., 2021). Thus, we speculated that the action mechanism of strain LB-1 against *B. cinerea* could be ascribed to certain antifungal substances damaging cell wall/membrane integrity by inhibiting the expression of related genes. It is worth noting that phenolic substances, including ferulic acid, chlorogenic acid, ouabain, and 1-caffeoylquinic acid, maybe the main antifungal components of strain LB-1 (Table 1).

With the destruction of cell walls and membranes, pathogens have developed different strategies to cope with adverse and toxic conditions. ABC transporters have been confirmed to be associated with fungicide resistance in fungal species (Winski et al., 2022). For example, previous data from transcriptomic analysis of *B. cinerea* showed that ABC transporter was highly induced during self-detoxification processes (Samaras et al., 2021; Wang et al., 2022a), fitting well with our data, the expression of seven genes encoding ABC transporters (*ABCB1, ABCC1*, and *ABCG2*) were induced in *B. cinerea* by LB-1 (Figure 4A, Supplementary Table 4). Meanwhile, the expression patterns of two genes encoding ABCB (*BCIN_01g03740*) and ABCG (*BcatrA*) transporters were validated by qRT-PCR (Figure 5). Among these transport proteins, ABCB1 and ABCG2 were well-known for transportation xenobiotics, such as fungicides and secondary metabolites (Viglas and Olejníková, 2021; Nordgren and Fransen, 2014), indicating that the extra-transport of the antifungal substances produced by LB-1 was enhanced by activated transporters in *B. cinerea*.

**Figure 6:**
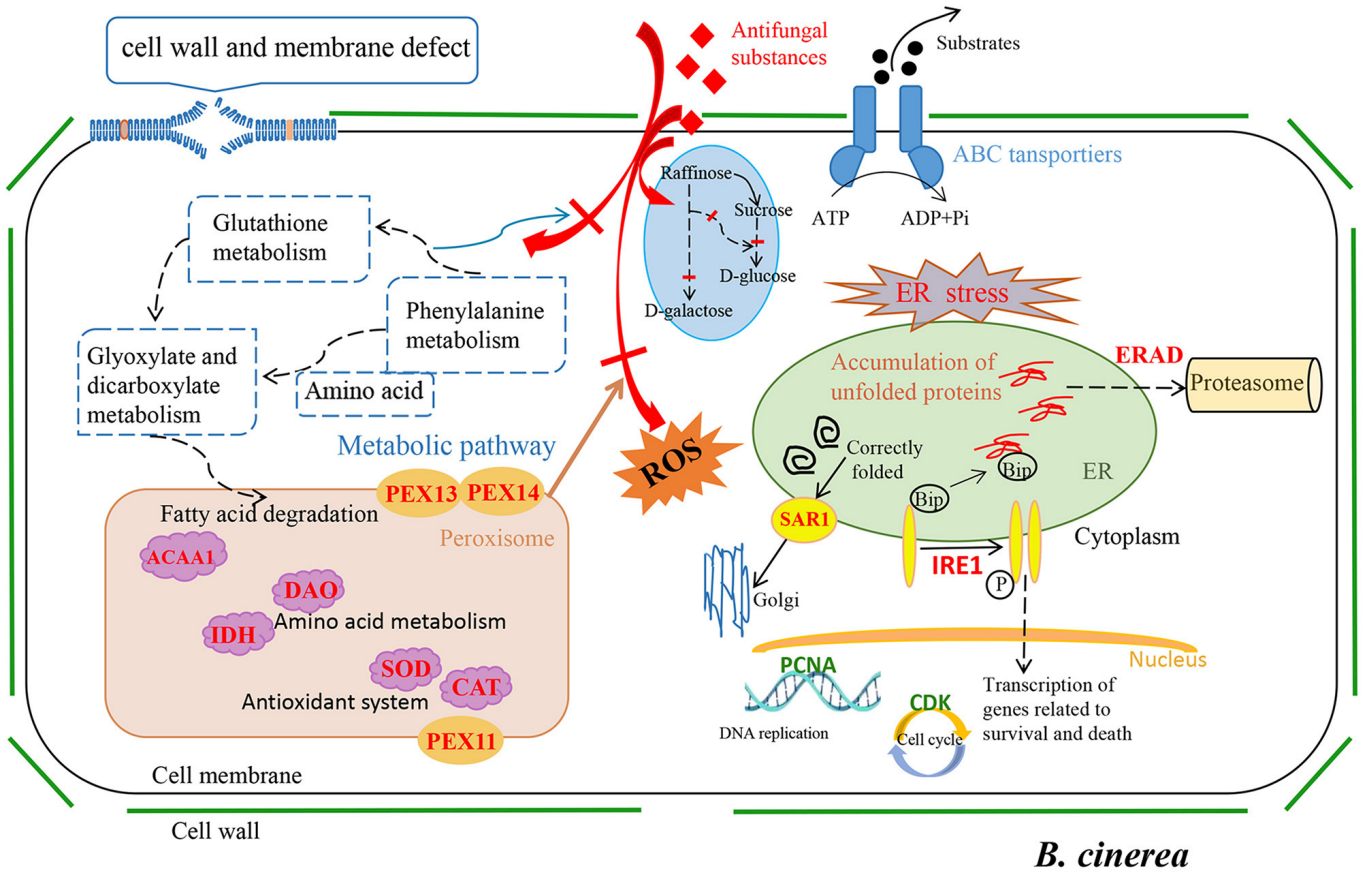
Proposed inhibition mechanisms for antifungal metabolites of LB-1 against *Botrytis cinerea*.

As we all know, environmental factors, such as fungicides and heat exposure, markedly increase ROS production and elicit oxidative stress in fungal cells (Wang et al., 2022b). Peroxisomes play an important role in redox homeostasis (Nordgren and Fransen, 2014). Liu et al. found upregulation of peroxisomes during their study of using 2-phenylethanol, isolation of *Kloeckera apiculata*, to inhibit *Penicillium molds* (Liu et al., 2014). Meng et al. also found similar findings during research on biocontrol *Clonostachys rosea* against *B. cinerea* (Meng et al., 2022). In this study, the proliferation of peroxisomes in *B. cinerea* was increased (Figure 4A, Supplementary Table 5), and multiple peroxisomal-related enzymes, especially some antioxidative enzyme genes, such as CAT (*Bccat5, BccatA*, and *Bccat6*; Supplementary Table 5), were significantly upregulated. Moreover, GSH was also involved in defending against intracellular oxidative damage (Noctor et al., 2002). In current study, six GST genes (*Bcgst3, Bcgst14, Bcgst21, Bcgst5, Bcgst1, Bcgst22, Bcgst26*, and *Bcgst6*) related to GSH metabolism were upregulated by LB-1 (Figure 4A; Supplementary Table 6). Among these genes, *Bccat5, BccatA*, and *Bcgst1* were validated by qRT-PCR (Figure 5); of which, the *Bcgst1* (Prins et al., 2000) has been confirmed to be closely related to the detoxification of ROS. Therefore, these findings indicated that strain LB-1 led to oxidative stress of *B. cinerea* (Figure 6).

Meanwhile, our results showed that strain LB-1 interfered with the ER function of *B. cinerea*, resulting in the accumulation of unfolded or misfolded proteins, which triggers a well-conserved pathway called the unfolded protein response (UPR) pathway (improve correct protein folding or degradation of misfolded proteins) to mitigate the ERS and promoted cell surviving (Figure 4A; Figure 6). However, if repair or degradation is inadequate, cells will activate apoptotic pathways and induce apoptosis under sustained stress stimulation (Krshnan et al., 2022). Moreover, the ERS receptor protein–IRE1 (*Bcire1*), as well as its related signaling pathway in the UPR, was activated (Figure 6; Supplementary Table 7). It is worth noting that the IRE1 signaling pathway can induce programmed cell death (Krshnan et al., 2022). These pathways have been reported widely in the death process of pathogens induced by antifungal substances (Wang et al., 2022b; Zhang et al., 2023c). Here, these data indicate that ERS-induced programmed cell death might be a key pathway for LB-1 to inhibit the growth of *B. cinerea*.

In this study, similar to some microorganisms under ethanol (Tibocha-Bonilla et al., 2022) or linoleic (Senizza et al., 2020) stress, *B. cinerea* responses to LB-1 stress also include various modifications in amino acid metabolism, such as tyrosine and tryptophan (Figure 4A). Amino acids, cofactors, phenylalanine, or pyruvate play important roles in the physiological metabolism of microorganisms, controlling many biological functions such as cell division, cell wall formation, cell growth, and metabolism. The contribution of their metabolic changes in pathogens to the antifungal effect of LB-1 requires further research.

In addition to these stress response pathways, LB-1 hindered carbohydrate metabolism linked to glycolysis (Figure 4B), thereby impeding growth in *B. cinerea*, and this antimicrobial pathway was also discovered in the inhibition of *Alternaria alternata* by carvacrol (Zhao et al., 2024). Additionally, multiple genes related to cell cycle, meiosis, and DNA replication pathways in *B. cinerea* were significantly downregulated, such as cyclin-dependent kinases (CDKs; *Bccks1*, and *Bccdc28*; Figure 4B, Supplementary Table 8; Supplementary Table 7). Similar to our findings, the use of antifungal substances to inhibit pathogens could directly inhibit fungal growth by downregulating these pathways, such as linalool to *Aspergillus flavus* (Li et al., 2022), C17 mycosubtilin to *V. dahliae* (Zhang et al., 2023d), antifungal substances produced by *Xenorhabdus bovienii* to *Fusarium solani* (Wang et al., 2022c). Antifungal substances could damage the DNA and induce autophagy of pathogens (Li et al., 2022; Goldman et al., 2002). In this study, the ability of mismatch repairs, such as nucleotide excision repair and base excision repair, was significantly inhibited (Figure 4B; Supplementary Tables 8–10), suggesting that the DNA damage was not being effectively repaired in *B. cinerea*. These results indicated that metabolites produced by LB-1 may directly limit or even block the process of DNA replication and cell proliferation, resulting in a serious impact on the growth of *B. cinerea* (Figure 6).

## Conclusion

In this study, the antifungal metabolites and their action mechanism of strain LB-1 against *B. cinerea* were investigated. A total of 45 metabolites, such as ouabain, ferulic acid, chlorogenic acid, spermidine, stachydrine, and stearic acid, were considered to be key antifungal substances. The action mechanism of strain LB-1 against *B. cinerea* could be described as damaging cell wall/cell membrane integrity, interfering with carbohydrate metabolism, leading to ERS, inhibiting DNA replication and meiosis, and blocking the cell cycle, which has changed the expression of associated genes and then induced apoptosis of *B. cinerea* (Figure 6).

## Data Availability

The datasets presented in this study can be found in online repositories. The names of the repository/repositories and accession number(s) can be found at: https://www.ncbi.nlm.nih.gov/, PRJNA953188.
