# Supplementary material for: Antifungal metabolites of biocontrol stain LB-1 and their inhibition mechanism against Botrytis cinerea
Source: Front Microbiol. 2024 Sep 4;15:1444996. doi: 10.3389/fmicb.2024.1444996 (PMC11409189; doi:10.3389/fmicb.2024.1444996)
Supplement: Supplementary file 1 [file Table_1.DOCX]

Fig. S1 The pie chart shows the chemical classification of 71 the possible antifungal metabolites

Fig. S2 Pearson correlation between samples


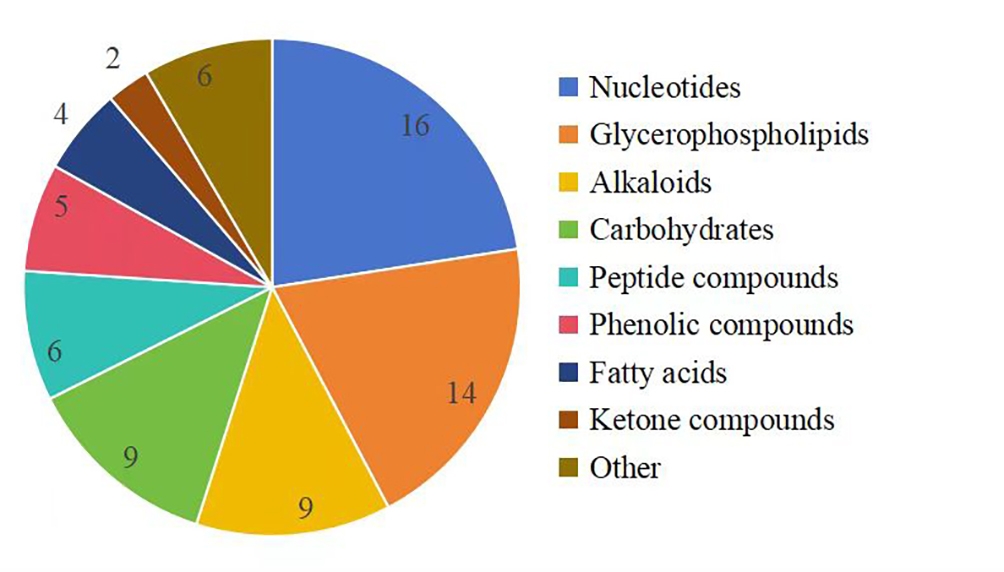


Fig. S1


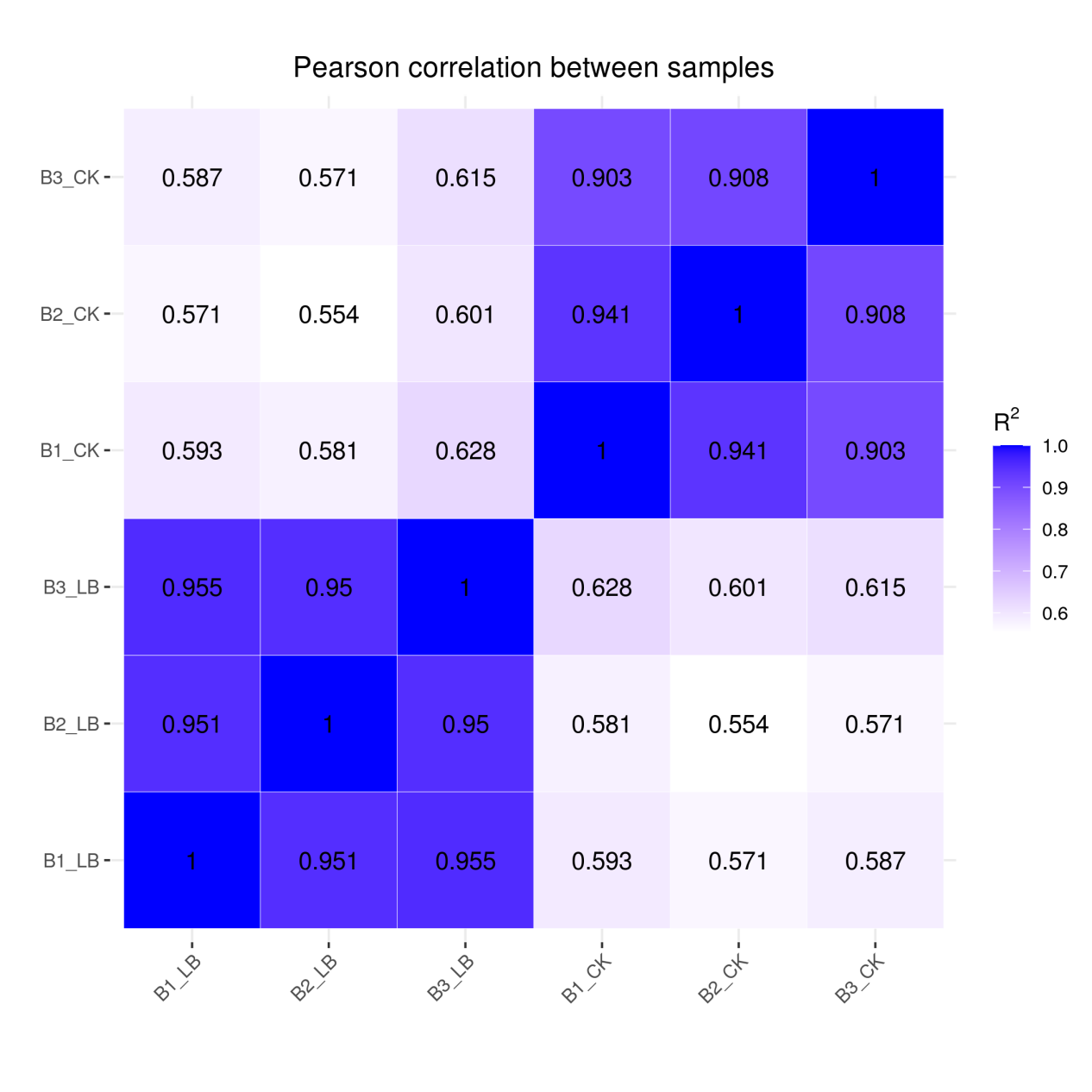


Fig. S2
