# Supplementary material for: Antifungal metabolites of biocontrol stain LB-1 and their inhibition mechanism against Botrytis cinerea
Source: Front Microbiol. 2024 Sep 4;15:1444996. doi: 10.3389/fmicb.2024.1444996 (PMC11409189; doi:10.3389/fmicb.2024.1444996)
Supplement: Supplementary file 2 [file Table_2.DOCX]

Tables S1 Gradient elution process of Vanquish UHPLC-MS/MS

Tables S2 Mass parameters and collision energy in Q Exactive™ HF

Table S3 Primers of 20 DEGs used for gene expression analysis by RT-qPCR

Table S4 Genes related to ABC transporters

TableS5 Genes related to peroxisomes

TableS6 Genes related to glutathione metabolism

TableS7 Genes related to endoplasmic reticulum stress

Table S8 Genes related to cell cycle

Table S9 Genes related to meiosis

Table S10 Genes related to DNA replication

**Tables S1 Gradient elution process of Vanquish UHPLC-MS/MS**

| Time (min) | Flow rate (mL/min) | Mobile phaseA(% ) | Mobile phase B (%) |
| --- | --- | --- | --- |
| 0 | 0.2 | 98 | 2 |
| 1.5 | 0.2 | 98 | 2 |
| 3 | 0.2 | 15 | 2 |
| 10 | 0.2 | 0 | 100 |
| 10.1 | 0.2 | 98 | 2 |
| 11 | 0.2 | 98 | 2 |
| 12 | 0.2 | 98 | 2 |

Mobile phase A: ultrapure water (0.1% formic acid) and mobile phase B: methanol in ESI-positive;

Mobile phase A: ultrapure water (5 mM ammonium acetate, pH: 9.0) and mobile phase B: methanol in ESI-negative modes.

**Tables S2 Mass parameters and collision energy in Q Exactive™ HF**

| Mass | Parameters |
| --- | --- |
| M/Z | 100-1500 |
| Sheath gas flow rate | 35psi |
| Aux Gas flow rate | 10L/min |
| Capillary Temp | 320℃ |
| IonSpray Voltage Floating (ESI+) (V) | 3.5kV |
| S-lens RF level | 60 |
| Aux gas heater temp | 350℃ |
| Polarity | positive, negative |

**Table S3 Primers of 15 DEGs used for gene expression analysis by RT-qPCR.**

| Gene ID | gene_name | Primer | Sequence (5’-3’) |
| --- | --- | --- | --- |
| BCIN_03g01920 | *Bccat5* | up | CCAACCCCAACTACCCATCT |
|  |  | down | TCCCACAGACCCTTAGCTTG |
| BCIN_07g01430 | *Bcsec62* | up | GAAACCACACAGCGAAACCT |
|  |  | down | CAGCCCATCCATAAATCGCC |
| BCIN_04g02090 | *Bcufd2* | up | TTAAAGTGGCACGGGATCCT |
|  |  | down | GACAGAACACCTTCGATGGC |
| BCIN_01g02660 | *Bcire1* | up | GGCTCAAGGTGGTGAAAAGG |
|  |  | down | CAACTAGGATGTTCTGCGGC |
| CIN_01g03740 | *CIN_01g03740* | up | AAGTGTAGGCTCCAGTGGAC |
|  |  | down | GTCCAAGATAAGCACAGCGG |
| BCIN_11g04460 | *BcatrA* | up | GATGCTACACGAAACCGACC |
|  |  | down | AGACTGTGTAAGGTGACCGG |
| BCIN_10g00740 | *Bcgst1* | up | GATCCACACAACATCGGAGC |
|  |  | down | CCTGCTACCCTTGATCTCGT |
| BCIN_06g01180 | *BccatA* | up | CCCGTTCTAAAGCCAGCATC |
|  |  | down | GACGACACGCTCAGGAATTC |
| BCIN_09g02790 | *BCIN_09g02790* | up | GTGTTACCCCTACCAAGCCT |
|  |  | down | AAGCCAATACACCCTCGACA |
| BCIN_02g07440 | *BCIN_02g07440* | up | GCCCTCTACGAATCCATCCA |
|  |  | down | TGGGTTGGGATGTTTTGCAG |
| BCIN_15g05270 | *Bcpie2* | up | CCTTCCACTCCCCTCAACTT |
|  |  | down | AACAACGTAGGCAAGGAGGA |
| BCIN_01g09530 | *BCIN_01g09530* | up | AAGGCCGCAAAATCACCATT |
|  |  | down | CCAAAATACTCCTCGCTGCC |
| BCIN_12g06300 | *BCIN_12g06300* | up | CGAGACCTCATTCAACGCAG |
|  |  | down | CAACGGAAACAGTGATGGCA |
| BCIN_05g01660 | *BCIN_05g01660* | up | TCCTCATACGTCGGTTCCTG |
|  |  | down | GCCCTCGGTCTTGAAATTCC |
| BCIN_06g03790 | *BCIN_06g03790* | up | AGTCAACTCTCAATGCCGGA |
|  |  | down | ACACCACCGGAGTAAGAAGG |

**Table S4 Genes related to ABC transporters**

| Subfamily | **gene_ID** | **gene_name** | **gene_description** | **log2FC** |
| --- | --- | --- | --- | --- |
| ABCB | BCIN_01g03740 | *BCIN_01g03740* | sp\|B2KWH4\|ABC1_AJECA ABC transporter 1 | 2.55 |
|  | BCIN_16g03560 | *BCIN_16g03560* | sp\|F2Q5G0\|MDR4_TRIEC ABC multidrug transporter MDR2 | 1.71 |
|  | BCIN_12g00700 | *BCIN_12g00700* | sp\|A0A059JK44\|MDR4_TRIIM ABC multidrug transporter MDR2 | 1.52 |
| ABCC | BCIN_01g00180 | *BCIN_01g00180* | sp\|Q4WT65\|ABCB_ASPFU ABC multidrug transporter B | 2.27 |
| ABCG | BCIN_02g03630 | *Bcpdr11* | sp\|P41820\|BFR1_SCHPO Brefeldin A resistance protein | 2.80 |
|  | BCIN_11g04460 | *BcatrA* | sp\|B8NDS8\|ATRF_ASPFN ABC multidrug transporter atrF | 5.78 |
|  | BCIN_04g02930 | *Bmr5* | sp\|Q4WFQ4\|ABCH_ASPFU ABC multidrug transporter H | 4.38 |

**TableS5 Genes related to peroxisomes**

| KO | gene_**ID** | gene_name | gene_description | log2FC |
| --- | --- | --- | --- | --- |
| PEX13 | BCIN_05g05280 | *Bcpex13* | sp\|Q92266\|PEX13_PICPA Peroxisomal membrane protein PEX13 | 2.00 |
| PEC1 | BCIN_16g03900 | *BCIN_16g03900* | sp\|Q05871\|ECI1_YEAST 3,2-trans-enoyl-CoA isomerase | 2.73 |
| NUDT12 | BCIN_15g02650 | *Bcnpy1* | sp\|Q9Y7J0\|NPY1_SCHPO NAD-capped RNA hydrolase | 2.51 |
| CRAT | BCIN_02g02420 | *BCIN_02g02420* | sp\|P80235\|CACM_YEAST Putative mitochondrial carnitine O-acetyltransferase | 3.10 |
| IDH | BCIN_09g00980 | *BCIN_09g00980* | sp\|P79089\|IDHP_ASPNG Isocitrate dehydrogenase [NADP], mitochondrial | 1.54 |
| PIPOX | BCIN_06g00740 | *Bcdao5* | sp\|B8NI25\|IMQH_ASPFN Amino acid oxidase imqH | 1.50 |
| DAO | BCIN_13g00810 | *Bcdao3* | sp\|Q99042\|OXDA_TRIVR D-amino-acid oxidase | 1.59 |
| CAT | BCIN_03g01920 | *Bccat5* | sp\|P55306\|CATA_SCHPO Catalase | 1.75 |
| CAT | BCIN_06g01180 | *BccatA* | sp\|P55304\|CATA_BOTFU Catalase A | 3.43 |
| CAT | BCIN_05g04580 | *Bccat6* | sp\|P55306\|CATA_SCHPO Catalase | 2.43 |
| EPHX2 | BCIN_15g00230 | *BCIN_15g00230* | PF00561:alpha/beta hydrolase fold && PF00561:alpha/beta hydrolase fold | 2.41 |
| EPHX2 | BCIN_13g00770 | *BCIN_13g00770* | sp\|Q7SHI0\|SRDG_NEUCR Epoxide hydrolase | 2.54 |

**TableS6 Genes related to glutathione metabolism**

| gene_id | gene_name | gene_description | log2FC |
| --- | --- | --- | --- |
| BCIN_11g00390 | *Bcgst3* | sp\|P77526\|YFCG_ECOLI Disulfide-bond oxidoreductase YfcG | 4.60 |
| BCIN_07g03430 | *Bcgst14* | sp\|Q9X4F7\|MAAI_RHIME Maleylacetoacetate isomerase | 5.09 |
| BCIN_07g03470 | *Bcgst21* | sp\|J4UHQ8\|OPS6_BEAB2 Glutathione S-transferase-like protein OpS6 | 7.20 |
| BCIN_14g03160 | *Bcgst5* | sp\|B3FWR8\|HPM2_HYPSB Glutathione S-transferase hmp2 | 8.60 |
| BCIN_14g01940 | *BCIN_14g01940* | PF00248:Aldo/keto reductase family | 3.54 |
| BCIN_10g00740 | *Bcgst1* | sp\|Q0CCY0\|GEDE_ASPTN Glutathione S-transferase-like protein gedE | 5.01 |
| BCIN_01g11420 | *Bcgst22* | -- | 3.53 |
| BCIN_01g08520 | *Bcprx4* | sp\|P34227\|PRX1_YEAST Peroxiredoxin PRX1, mitochondrial | 3.34 |
| BCIN_14g03920 | *Bcgsh1* | sp\|Q8X0X0\|GSH1_NEUCR Glutamate--cysteine ligase | 2.06 |
| BCIN_16g03610 | *Bcgst26* | PF01124:MAPEG family | 2.20 |
| BCIN_09g00980 | *BCIN_09g00980* | sp\|P79089\|IDHP_ASPNG Isocitrate dehydrogenase [NADP], mitochondrial | 1.54 |
| BCIN_09g00820 | *Bcgst6* | sp\|Q4WQZ2\|TPCF_ASPFU Glutathione S-transferase-like protein tpcF | 4.10 |

**TableS7 Genes related to endoplasmic reticulum stress**

| KO | gene_id | gene_name | gene_description | log2FC |
| --- | --- | --- | --- | --- |
| Sec62/63 | BCIN_07g01430 | *Bcsec62* | sp\|Q99161\|SEC62_YARLI Translocation protein SEC62 | 2.26 |
| UbcH5 | BCIN_02g04920 | *BCIN_02g04920* | sp\|O74196\|UBC1_COLGL Ubiquitin-conjugating enzyme E2-16 kDa | 1.87 |
| Doa10 | BCIN_07g07070 | *Bcssm4* | sp\|O60103\|DOA10_SCHPO ERAD-associated E3 ubiquitin-protein ligase doa10 | 4.00 |
| CHIP | BCIN_15g04190 | *BCIN_15g04190* | sp\|Q54IP0\|DNJC7_DICDI DnaJ homolog subfamily C member 7 homolog | 2.88 |
| sHSF | BCIN_15g02550 | *BCIN_15g02550* | sp\|O14368\|HSP16_SCHPO Heat shock protein 16 | 3.69 |
| Ubc6/7 | BCIN_01g04790 | *Bcubc6* | sp\|O42646\|UBC6_SCHPO Ubiquitin-conjugating enzyme E2 6 OS=*Schizosaccharomyces pombe* (strain 972 / ATCC 24843) | 2.63 |
| NEF | BCIN_16g02010 | *Bcfes1* | sp\|Q4I624\|FES1_GIBZE Hsp70 nucleotide exchange factor FES1 | 4.37 |
| Otu1 | BCIN_02g06960 | *Bcotu1* | sp\|Q29FC9\|OTU1_DROPS Ubiquitin thioesterase OTU1 | 2.37 |
| Hsp90 | BCIN_10g00300 | *BCIN_10g00300* | sp\|O43109\|HSP90_PODAS Heat shock protein 90 homolog | 3.10 |
| Ufd2 | BCIN_04g02090 | *Bcufd2* | sp\|Q9HE05\|UFD2_SCHPO Ubiquitin conjugation factor E4 | 2.18 |
| Derlin | BCIN_03g09290 | *BCIN_03g09290* | PF04511:Der1-like family | 2.16 |
| Sec62/63 | BCIN_10g03970 | *Bcsec63* | sp\|Q9HGN7\|SEC63_SCHPO Translocation protein sec63 | 1.92 |
| DSK2 | BCIN_05g00330 | *Bcdsk2* | sp\|P48510\|DSK2_YEAST Ubiquitin domain-containing protein DSK2 | 1.79 |
| RAD23 | BCIN_10g05180 | *Bcrad23* | sp\|O74803\|RHP23_SCHPO UV excision repair protein rhp23 | 1.66 |
| Png1 | BCIN_01g01080 | *Bcpng1* | sp\|Q4IR87\|PNG1_GIBZE Protein PNG1 | 1.55 |
| Ubc6/7 | BCIN_08g04560 | *BCIN_08g04560* | sp\|Q9Y385\|UB2J1_HUMAN Ubiquitin-conjugating enzyme E2 J1 | 1.00 |
| Ero1 | BCIN_05g00150 | *Bcero1* | sp\|Q7SEY9\|ERO1_NEUCR Endoplasmic reticulum oxidoreductin-1 | 1.82 |
| IRE1 | BCIN_01g02660 | *Bcire1* | sp\|O94537\|IRE1_SCHPO Sensor for unfolded proteins in the ER ire1 | 1.70 |

**Table S8 Genes related to cell cycle**

| gene_id | gene_name | gene_description | log2FC |
| --- | --- | --- | --- |
| BCIN_07g04250 | *Bcorc4* | sp\|Q9Y794\|ORC4_SCHPO Origin recognition complex subunit 4 | -2.92 |
| BCIN_15g03130 | *Bccdc23* | sp\|O94556\|APC8_SCHPO Anaphase-promoting complex subunit 8 | -2.32 |
| BCIN_02g04000 | *Bcmps1* | sp\|P54199\|MPS1_YEAST Serine/threonine-protein kinase MPS1 | -2.12 |
| BCIN_02g05160 | *Bcycs4* | sp\|O94679\|CND1_SCHPO Condensin complex subunit 1 | -1.60 |
| BCIN_06g05900 | *Bcbub2* | sp\|P36618\|CDC16_SCHPO Cell division control protein 16 | -1.75 |
| BCIN_05g00160 | *Bccks1* | sp\|P08463\|CKS1_SCHPO Cyclin-dependent kinases regulatory subunit | -1.73 |
| BCIN_02g05150 | *Bccdc7* | sp\|P50582\|HSK1_SCHPO Cell cycle serine/threonine-protein kinase hsk1 | -1.84 |
| BCIN_07g02410 | *Bcmih1* | sp\|P30303\|MPIP_EMENI M-phase inducer phosphatase | -1.72 |
| BCIN_02g06170 | *Bcsmc4* | Bsp\|P41004\|SMC4_SCHPO Structural maintenance of chromosomes protein 4 | -1.55 |
| BCIN_03g02820 | *Bcorc2* | sp\|Q09142\|ORC2_SCHPO Origin recognition complex subunit 2 | -1.62 |
| BCIN_01g10610 | *Bcmcm7* | sp\|O75001\|MCM7_SCHPO DNA replication licensing factor mcm7 | -1.78 |
| BCIN_05g00810 | *Bccdc20* | sp\|P78972\|SLP1_SCHPO WD repeat-containing protein slp1 | -1.89 |
| BCIN_15g02990 | *BCIN_15g02990* | sp\|P41389\|MCM5_SCHPO DNA replication licensing factor mcm5 | -1.61 |
| BCIN_16g00490 | *Bccdc28* | sp\|P54119\|CDK1_AJECA Cyclin-dependent kinase 1 | -1.45 |
| BCIN_04g04220 | *Bcmad2* | sp\|O14417\|MAD2_SCHPO Mitotic spindle checkpoint component mad2 | -1.68 |
| BCIN_11g05760 | *Bcmcm4* | sp\|P29458\|MCM4_SCHPO DNA replication licensing factor mcm4 | -1.43 |
| BCIN_15g02010 | *Bcorc5* | PF13191:AAA ATPase domain\|PF14630:Origin recognition complex (ORC) subunit 5 C-terminus | -1.54 |
| BCIN_14g00280 | *BCIN_14g00280* | sp\|P41412\|RES2_SCHPO Cell division cycle-related protein res2/pct1 | -1.83 |
| BCIN_13g03760 | *BCIN_13g03760* | hypothetical protein | -2.24 |

**Table S9 Genes related to meiosis**

| gene_id | gene_name | gene_description | log2FC |
| --- | --- | --- | --- |
| BCIN_09g05240 | *BCIN_09g05240* | sp\|K0E3U9\|ECDD_ASPRU Major facilitator-type transporter ecdD | -3.76 |
| BCIN_07g04250 | *Bcorc4* | sp\|Q9Y794\|ORC4_SCHPO Origin recognition complex subunit 4 | -2.92 |
| BCIN_15g03130 | *Bccdc23* | sp\|O94556\|APC8_SCHPO Anaphase-promoting complex subunit 8 | -2.32 |
| BCIN_02g05150 | *Bccdc7* | sp\|P50582\|HSK1_SCHPO Cell cycle serine/threonine-protein kinase hsk1 | -1.84 |
| BCIN_03g02820 | *Bcorc2* | sp\|Q09142\|ORC2_SCHPO Origin recognition complex subunit 2 | -1.62 |
| BCIN_01g10610 | *Bcmcm7* | sp\|O75001\|MCM7_SCHPO DNA replication licensing factor mcm7 | -1.78 |
| BCIN_05g00810 | *Bccdc20* | sp\|P78972\|SLP1_SCHPO WD repeat-containing protein slp1 | -1.89 |
| BCIN_15g02990 | *BCIN_15g02990* | sp\|P41389\|MCM5_SCHPO DNA replication licensing factor mcm5 | -1.61 |
| BCIN_06g00150 | *BCIN_06g00150* | sp\|Q6MYX6\|QUTD_ASPFU Probable quinate permease | -1.90 |
| BCIN_04g04220 | *Bcmad2* | sp\|O14417\|MAD2_SCHPO Mitotic spindle checkpoint component mad2 | -1.68 |
| BCIN_15g02010 | *Bcorc5* | PF13191:AAA ATPase domain\|PF14630:Origin recognition complex (ORC) subunit 5 C-terminus | -1.54 |
| BCIN_13g03760 | *BCIN_13g03760* | PF05460:Origin recognition complex subunit 6 (ORC6) | -2.24 |
| BCIN_02g00680 | *Bcspo11* | sp\|P40384\|SPO11_SCHPO Meiotic recombination protein rec12 | -2.11 |

**Table S10 Genes related to DNA replication**

| gene_id | gene_name | gene_description | log2FC |
| --- | --- | --- | --- |
| BCIN_07g00130 | *Bcpri1* | sp\|O14215\|PRI1_SCHPO DNA primase small subunit | -1.78 |
| BCIN_08g00460 | *Bcpri2* | sp\|Q8NIZ4\|PRI2_NEUCR Probable DNA primase large subunit | -1.85 |
| BCIN_01g10610 | *Bcmcm7* | sp\|O75001\|MCM7_SCHPO DNA replication licensing factor mcm7 | -1.78 |
| BCIN_12g01680 | *BCIN_12g01680* | hypothetical protein | -1.93 |
| BCIN_15g02990 | *BCIN_15g02990* | hypothetical protein | -1.61 |
| BCIN_02g02880 | *Bcrnh201* | sp\|Q9P5X8\|RNH2A_NEUCR Ribonuclease H2 subunit A | -2.14 |
| BCIN_02g00920 | *Bcrfc3* | sp\|O74111\|RFC3_BLAAD Replication factor C subunit 3 | -1.52 |
